# Synthesis, Properties, and Applications of Novel Polymer-Based Gels

**DOI:** 10.3390/gels12020143

**Published:** 2026-02-03

**Authors:** Zhen Gao, Peng Zhang, Yuanxun Zheng

**Affiliations:** School of Water Conservancy and Transportation, Zhengzhou University, Zhengzhou 450001, China; gao-zhen@zzu.edu.cn (Z.G.); yxzheng@zzu.edu.cn (Y.Z.)

## 1. Introduction

Polymer gels, as a class of soft materials with three-dimensional cross-linked networks, have garnered escalating attention in recent decades due to their exceptional capacity to absorb and retain solvents, coupled with tunable mechanical, chemical, and responsive properties [1]. These unique characteristics enable polymer gels to bridge the gap between traditional rigid materials and flexible substrates, finding widespread applications across diverse fields ranging from construction and transportation to biomedicine and environmental engineering [2]. In recent years, advances in synthesis technologies—such as enzyme-induced precipitation, alkali activation, and composite modification—have expanded the functional boundaries of polymer gels, allowing for precise control over their microstructure, reactivity, and performance [3]. Simultaneously, the growing emphasis on sustainability has driven the development of gel systems derived from industrial by-products, waste materials, and bio-based precursors, aligning with global efforts toward carbon neutrality and resource recycling [4].

The versatility of polymer gels is reflected in their diverse functional adaptations. For instance, in construction and geotechnical engineering, gel composites have been developed to enhance the freeze–thaw resistance of cementitious materials, stabilize weak soils, and improve the durability of concrete structures against chloride ion penetration [5]. In transportation infrastructure, gel-modified asphalt mixtures and anti-skid wear layers have emerged as effective solutions to address pavement aging, ice formation, and structural degradation [6]. The biomedical sector has witnessed the development of self-healing hydrogels for strain sensing and molecularly imprinted gel sensors for rapid biomarker detection, leveraging the biocompatibility and responsiveness of gel networks [7]. Additionally, environmental applications of polymer gels include dust suppression in silt soils and the valorization of waste materials (e.g., river sediments and rubber particles) into high-performance gel composites [8].

Cutting-edge research on polymer gels is characterized by interdisciplinary integration, combining insights from materials science, chemistry, engineering, and biology to address complex real-world challenges [9]. Key advancements include the development of multifunctional gels with synergistic properties (e.g., self-healing, high stretchability, and sensitivity), the optimization of synthesis processes for improved sustainability, and the exploration of novel application scenarios in harsh or specialized environments [10]. To capture these latest developments and foster knowledge exchange, the *Gels* journal presents this Special Issue, “Synthesis, Properties, and Applications of Novel Polymer-Based Gels,” featuring eleven high-quality contributions that showcase the state of the art in polymer gel research. These studies span synthesis innovations, performance optimization, and practical applications, reflecting the dynamic and interdisciplinary nature of the field. In the following section, we provide an overview of each contribution, highlighting the study’s aims, key findings, and relevance to the broader context of gel materials and technology.

## 2. Overview of the Papers Published in This Special Issue

The paper “A State-of-the-Art Review on the Freeze–Thaw Resistance of Sustainable Geopolymer Gel Composites: Mechanisms, Determinants, and Models” conducts a comprehensive and in-depth review on the freeze–thaw (F-T) resistance of geopolymer gel composites (GCs), a topic of great significance for promoting the practical application of geopolymer materials. The authors focus on geopolymers, a sustainable and low-carbon gel binder regarded as a potential alternative to cement, and emphasize that F-T resistance is a crucial indicator for evaluating the durability of GCs, as it exerts a profound impact on the service life of structures. To clarify the intrinsic principles of F-T damage in geopolymers, the authors systematically synthesize several commonly used theories related to F-T damage, while also meticulously elaborating on the key factors that influence the F-T resistance of GCs, including raw materials, curing conditions, and modified materials. The review findings indicate that the F-T resistance of GCs can be significantly enhanced by adopting high-calcium-content precursors, mixed alkali activators, and rubber aggregates; appropriately increasing the curing temperature also improves F-T resistance, especially for GCs made with low-calcium-content precursors. Among modified materials, most fibers and nano-materials remarkably boost the F-T resistance of GCs, whereas air-entraining agents have a negligible effect on this property. Furthermore, the authors summarize evaluation and prediction models for F-T damage of GCs, encompassing empirical models and machine learning models, and note that machine learning-based models exhibit higher predictive accuracy compared to empirical ones. The review’s findings are highly relevant for the construction and materials science industries, promoting a deeper understanding of the factors affecting the F-T resistance of GCs and their underlying mechanisms, thus providing a solid foundation for both engineering practice and academic research in this field.

The second article, “Valorization of River Sediments in Sustainable Cementitious Gel Materials: A Review of Characteristics, Activation, and Performance” explores the high-value utilization of river sediments as alternative raw materials for sustainable cementitious gel materials, addressing the dual goals of resource recycling and carbon reduction in the construction industry. The authors highlight that river sediments, abundant in supply and rich in silica-alumina components, have emerged as promising substitutes for traditional raw materials in cementitious systems, aligning with global sustainability initiatives. To conduct a comprehensive review, they performed a systematic literature search on Web of Science and Google Scholar using keywords such as “river sediment,” “cementitious materials,” “activation,” and “pozzolanic activity,” covering publications up to July 2025, and employed the Connected Papers citation network tool to ensure the inclusion of emerging studies. The review systematically examines the properties of river sediments from diverse regions, analyzes activation and modification techniques (including alkali/acid activation, thermal calcination, and mechanical milling), and evaluates their applications in various cementitious systems by comparing mix design models to clarify the effects of replacing fine aggregates, coarse aggregates, and cement on workability, strength, and durability. Multi-scale characterization via XRD, FTIR, and TG-DSC reveals the underlying mechanisms, including the formation of C-S-H and C-A-S-H gels, pore refinement, and densification of the interfacial transition zone. Three key findings are emphasized: (1) moderate sediment replacement (20–30%) enhances strength without compromising flowability; (2) alkali–water glass activation and calcination at 600–850 °C effectively improve pozzolanic activity; and (3) combining the minimum paste thickness theory with additives like water reducers, fibers, or biochar enables the design of high-performance, low-carbon concrete. This work demonstrates a critical trend in sustainable construction materials: leveraging industrial and natural waste (river sediments) in cementitious gels can reduce reliance on traditional raw materials, lower carbon emissions, and promote resource recycling. The study’s implications extend to greener building practices and the development of low-CO_2_ cementitious materials, providing a comprehensive theoretical foundation and technical pathway for the sustainable valorization of river sediments.

The third article, “Performance and Mechanism Analysis of an Anti-Skid Wear Layer of Active Slow-Release Ice–Snow Melting Modified by Gels” delves into the development of sustainable pavement materials for winter maintenance, addressing the long-standing challenge of balancing large-scale upkeep efficiency and driving safety, particularly in the application of active slow-release ice-melting materials. The authors set out to enhance the ice-melting capacity of pavement through controlled salt release, proposing a gel-modified salt-storing ceramsite asphalt mixture as an innovative solution. They conducted systematic experiments by replacing conventional coarse aggregates with salt-storing ceramsite in SMA-10 graded mixtures (with replacement ratios ranging from 0% to 80%), comprehensively evaluating the mixture’s mechanical performance and de-icing functionality. The key experimental results reveal that a 40% salt-storing ceramsite content optimizes the mixture’s high-temperature stability while maintaining acceptable low-temperature performance and water resistance—critical balances for winter pavement durability. Microstructural analysis further elucidates the underlying mechanisms: the silicone–acrylic emulsion forms a hydrophobic film on the surface of ceramsite, enabling uniform salt distribution and sustained release, while 10% gel modification achieves effective salt retention and controlled release through pore-structure regulation. A practical salt-storing ceramsite content range of 40–60% is established for winter pavement applications, addressing the core need for durable, effective snow-melting asphalt surfaces. This study represents a significant advancement in winter pavement technology, as it integrates gel modification with salt-storing ceramsite to realize active slow-release ice-melting, avoiding the trade-offs between mechanical performance and de-icing efficiency that plague traditional materials. The findings provide valuable insights for the design of long-lasting, safe winter pavement surfaces, offering a reliable technical solution to improve driving safety in cold regions while optimizing maintenance efficiency.

In “Bio-Gel Formation Through Enzyme-Induced Carbonate Precipitation for Dust Control in Yellow River Silt”, address an environmental challenge in silt management using gel-enhanced bio-cementation technology. Yellow River silt is prone to dust emission, which poses ecological and health risks, and traditional dust control methods often lack durability or environmental sustainability. This study explored a novel hydrogel-modified enzyme-induced carbonate precipitation (EICP) approach to form reinforced bio-cemented crusts, aiming to enhance the compressive strength and resistance to wind–rain erosion of silt soils. The authors optimized the cementation solution—adopting equimolar concentrations of urea and CaCl_2_ at 1.25 mol/L to improve the uniformity of CaCO_3_ precipitation—and determined a spraying volume of 4 L/m^2^ (applying urea-CaCl_2_ solution first, followed by urease solution) as the optimal parameter. Experimental results showed this setup yielded a 14.9 mm thick hybrid gel-CaCO_3_ crust with a compressive strength exceeding 752 kPa. Mechanistic analysis via SEM confirmed the synergistic interaction between CaCO_3_ crystals and the gel matrix: the hydrogel network acted as a nucleation template, enhancing crystal bridging and pore-filling efficiency, while XRD analysis validated the formation of a stable gel-CaCO_3_ composite structure. The treated silt exhibited superior resistance to wind–rain erosion and mechanical wear compared to untreated samples. By establishing a positive correlation between gel-modified EICP and enhanced dust suppression performance, this work highlights a novel application of bio-gel composites in environmental engineering—one that enables sustainable dust mitigation in silt soils without relying on harsh chemicals. It underscores cross-disciplinary innovation where insights from bio-cementation and polymer gel technology are integrated to solve practical ecological challenges in silt management.

In “Self-Healing and Tough Polyacrylic Acid-Based Hydrogels for Micro-Strain Sensors”, the authors delve into the development of advanced hydrogel materials for smart sensing applications in bioengineering and intelligent systems. Self-healing hydrogels are widely recognized for their potential in these fields, yet they face a critical challenge: balancing excellent self-healing ability with sufficient mechanical strength. To address this bottleneck, the authors set out to develop a self-healing hydrogel with superior stretchability, employing a novel strategy of embedding a combination of hydrogen bonding and dynamic metal coordination interactions into a covalent polyacrylic acid (PAA) matrix. These interactions are introduced by three key components: modified fenugreek galactomannan, ferric ions, and lignin silver nanoparticles. Using this approach, Liu et al. successfully achieved a synergistic effect between covalent bonds and multiple non-covalent interactions, endowing the hydrogel with both high self-healing capacity and enhanced mechanical performance. In particular, the introduction of multiple energy dissipation mechanisms—most notably migrative dynamic metal coordination interactions-enabled the hydrogel to exhibit ultra-high stretchability, reaching up to 2000%. Furthermore, the incorporation of lignin silver nanoparticles and ferric ions endowed the hydrogel with excellent strain sensitivity, boasting a gauge factor of approximately 3.94, along with stable and repeatable resistance signals. When assembled into a flexible strain sensor, the hydrogel effectively detected subtle human motions and organ vibrations; it even demonstrated practical applicability by replacing conductive rubber in gaming controllers for real-time input functions. This development represents a significant innovation, as it overcomes the long-standing trade-off between self-healing ability and mechanical strength in hydrogels. The study provides a versatile strategy for designing multifunctional hydrogels, laying a foundation for their broader application in advanced sensing scenarios within bioengineering and intelligent systems.

“Study on Road Performance and Ice-Breaking Effect of Rubber Polyurethane Gel Mixture” delves into the development of high-efficiency, low-carbon, and environmentally friendly pavement materials for active snow melting and ice breaking, focusing on addressing critical challenges in road engineering. Inspired by the urgent need to solve a series of pressing issues-severe pavement temperature diseases, low efficiency and high loss of traditional ice-breaking methods, high occupancy rate of waste tires, and low utilization rate and insufficient durability of rubber particles, the authors set out to improve road service levels and ensure winter pavement safety. They adopted a systematic three-step research approach: (1) the optimization of rubber particle treatment using three different solutions-NaOH, NaClO, and KH550—along with the study of hydrophilic properties, surface morphology, and phase composition of rubber particles before and after optimization to determine the optimal treatment method; (2) the replacement of natural aggregates in polyurethane gel mixtures with the optimized rubber particles via the volume substitution method, coupled with the determination of optimal polyurethane gel dosage and molding-curing processes; and (3) the comparative analysis of the influence law of rubber particle content on the road performance of Rubber Polyurethane Gel Mixture (RPGM) through indoor tests, as well as the exploration of its ice-breaking effect. Using this approach, Chen et al. successfully obtained significant experimental findings: the contact angles of rubber particles treated with the three solutions were reduced by 22.5%, 30.2%, and 36.7%, respectively, with improved surface energy, reduced surface element types, and effective removal of surface impurities—among which the KH550 solution exhibited the most remarkable improvement effect. As rubber particle content increased from 0% to 15%, the dynamic stability of the mixture gradually increased (with a maximum increase of 23.5%), the maximum bending strain increased correspondingly, the residual stability first increased then decreased (with increased ranges of 1.4%, 3.3%, and 0.5%, respectively), and the anti-scattering performance improved—even with excessive rubber content, the scattering loss rate remained below 5%. Additionally, the fatigue life of polyurethane gel mixtures with 0%, 5%, 10%, and 15% rubber particles was 2.9 times, 3.8 times, 4.3 times, and 4.0 times higher than that of AC-13 asphalt mixture, demonstrating excellent anti-fatigue performance; the friction coefficient could be increased by up to 22.3% compared with ordinary asphalt mixtures. RPGM showed superior de-icing performance to traditional asphalt mixtures, with ice-breaking ability effectively enhanced as rubber particle content increased—though the ability weakened significantly when the ice layer thickness exceeded 9 mm. The ice-breaking mechanism was mainly achieved through the synergistic effect of stress coupling, thermal effect, and interface failure: under cyclic driving loads, the bonding performance of the ice–pavement interface was weakened, leading to loosening, breaking, and peeling of the ice layer to achieve efficient de-icing. This research represents a significant innovation, as it simultaneously resolves the problems of waste tire recycling and winter pavement safety, realizing the dual value of environmental protection and engineering practicality. The study provides a proof-of-concept for designing high-performance active snow-melting and ice-breaking pavement materials, where the integration of optimized rubber particles and polyurethane gel effectively improves both road durability and winter driving safety, offering valuable technical insights for practical engineering applications.

“A Study on the Performance of Gel-Based Polyurethane Prepolymer/Ceramic Fiber Composite-Modified Asphalt” delves into the development of composite-modified asphalt using gel-based materials, aiming to solve various problems in traditional roads and extend their service life—a critical focus as new road materials have become a research hotspot. Inspired by the unique and synergistic properties of polyurethane prepolymers (PUPs) and ceramic fibers (CFs)—where PUPs provide elasticity and gel-like behavior while CFs contribute to structural stability and high-temperature resistance—the authors set out to enhance asphalt performance by integrating these two materials. They adopted a systematic research approach combining optimization and comprehensive characterization: (1) the use of response surface methodology to investigate an optimized preparation scheme for PUP/CF composite-modified asphalt; and (2) the employment of multiple advanced testing techniques, including aging tests, dynamic shear rate (DSR) testing, bending rate (BBR) testing, multiple stress creep recovery (MSCR) testing, scanning electron microscopy (SEM), atomic force microscopy (AFM), and infrared spectroscopy (IR), to explore the aging performance, rheological properties, permanent deformation resistance, microstructure, and modification mechanism of the composite-modified asphalt. Using this approach, Guo et al. successfully determined the optimal preparation scheme: a PUP content of 7.4%, a CF content of 2.1%, and a shear time of 40 min. The experimental results indicate that the addition of PUP and CF significantly enhances the asphalt’s aging resistance; compared with single-CF-modified asphalt and base asphalt, the PUP/CF composite-modified asphalt exhibits superior high- and low-temperature rheological properties and stronger strain recovery capability. Microstructural analysis reveals that PUP forms a gel network structure in the material, effectively filling the gaps between CF and asphalt, enhancing interfacial bonding strength, and stabilizing overall performance. AFM microscopic morphology further shows that the PUP/CF composite-modified asphalt has more “honeycomb structures” than matrix asphalt and CF-modified asphalt, forming more structural asphalt and improving overall structural stability. This research represents a significant innovation, as it leverages the synergistic effect of PUP gel and CF to significantly improve both the macro and micro properties of asphalt-with the PUP forming a three-dimensional elastic gel network to enhance adhesion and deformation resistance. The study provides a proof-of-concept for designing high-performance modified asphalt materials, where the optimized formulation (7.4% PUP, 2.1% CF) effectively improves key asphalt properties (penetration ↓ 41.5%, softening point ↑ 6.7 °C, ductility ↑ 9%), demonstrating the great relevance of gel-based composites for asphalt modification in practical road engineering.

The paper “Study on the Adaptability of FBG Sensors Encapsulated in CNT-Modified Gel Material for Asphalt Pavement” explores an innovative application of modified gel materials in asphalt pavement strain monitoring. The authors develop a fiber Bragg grating (FBG) sensor encapsulated in carboxylated carbon nanotube (CNT-COOH)-modified gel material, which is designed to address the needs of prolonging asphalt pavement service life and reducing maintenance costs. While traditional FBG sensors may lack sufficient compatibility and durability when applied directly to asphalt pavement, the CNT-modified gel encapsulation is intended to enhance the sensor’s mechanical performance and adaptability to the pavement’s working environment. To optimize this encapsulation material, Guo et al. conduct tensile and bending tests to evaluate the effect of different CNT-COOH dosages on the mechanical properties of the gel material, ultimately determining the optimal dosage. They also use infrared spectroscopy and scanning electron microscopy to compare and analyze the infrared spectra and microstructure of carbon nanotubes before and after carboxyl functionalization, as well as the microstructure of the modified gel material. Laboratory test results show that the incorporation of CNT-COOH significantly improves the mechanical properties of the gel material: tensile strength, elongation at break, and tensile modulus increase by 36.2%, 47%, and 17.2%, respectively, while flexural strength, flexural modulus, and flexural strain increase by 89.7%, 7.5%, and 63.8%, respectively. Infrared spectrum analysis confirms the successful introduction of carboxyl (COOH) and hydroxyl (OH) groups on the surface of carbon nanotubes, and microstructure analysis reveals that carboxyl functionalization reduces CNT agglomeration, making the tensile section of the modified gel material rougher with obvious plastic deformation and improved toughness. Calibration experiments indicate that the strain and temperature sensitivity coefficients of the encapsulated sensor are 1.9864 pm/μm and 0.0383 nm/°C, which are 1.63 times and 3.61 times higher than those of bare FBG sensors. Applicability studies and ANSYS finite element analysis verify that the internal strain of asphalt rutting specimens changes linearly with external static load (with a fitting sensitivity of 0.0286 με/N), and simulation results are consistent with measured data, confirming the sensor’s effectiveness and monitoring accuracy. Dynamic load tests further reflect the internal strain change trend of asphalt mixture under external rutting load. The study’s findings are highly relevant for the road engineering industry, demonstrating how CNT-modified gel encapsulation can enhance FBG sensor performance, thereby providing a reliable technical solution for long-term strain monitoring of asphalt pavement.

“Quantum-Dot-Based Molecularly Imprinted Hydrogel for Rapid Detection of Homocysteine” delves into the development of advanced hydrogel-based detection materials, focusing on addressing the need for rapid and selective detection of homocysteine (Hcy)—a biomolecule whose elevated levels are associated with various pathological conditions including atherosclerosis, hypertension, and cardiovascular diseases. Inspired by the potential of quantum dots (QDs) for fluorescence-based detection and molecular imprinting technology for high selectivity, the authors set out to develop quantum-dot-based molecularly imprinted hydrogels (QD@MIHs) as a novel tool for rapid Hcy detection. They combined two key technologies: (1) the integration of L-cysteine-modified ZnS quantum dots (QDs) with highly selective molecular imprinting technology; and (2) the fabrication of QD@MIHs using a dual-functional monomer system (acrylamide and methacrylic acid) via surface coating of the Hcy molecularly imprinted polymer gel onto the QDs. Using this approach, Zhang et al. successfully developed the QD@MIHs and characterized their detection performance under optimal conditions. The experimental results showed that the response time of the QD@MIHs for Hcy detection was only 5 min, enabling rapid analysis. When the Hcy concentration ranged from 0.1 to 10.0 μM, the fluorescence quenching of the QD@MIHs exhibited a good linear relationship with Hcy concentration (R^2^ = 0.9972), with a corresponding detection limit as low as 0.027 μM, demonstrating high sensitivity. Additionally, the constructed QD@MIHs showed no significant response to other interfering substances, confirming the high selectivity of the prepared material. Practical sample analysis further validated the reliability of the method: the recovery rates of Hcy ranged from 94.34% to 104.1%, with relative standard deviations (RSD, n = 3) between 3.56% and 7.17%. This QD@MIH-based detection method represents a significant innovation, as it achieves rapid, selective, and sensitive Hcy detection with simple operation, overcoming the limitations of traditional detection methods that may be time-consuming or lack selectivity. The study provides a proof-of-concept for designing next-generation biomolecular detection tools—particularly for biomedical diagnostics and preventive-healthcare applications—where the integration of quantum dots and molecularly imprinted hydrogels enables efficient and reliable detection of key pathological markers.

“Polymer–Silicate Composite Gel Systems for Enhanced Chloride Resistance of Cement-Based Materials” delves into the development of advanced gel-based protective materials, focusing on addressing the critical issues of insufficient protection and poor durability of concrete during service. Inspired by the potential of silane and fluorocarbon resin emulsion to improve material protection performance, the authors set out to develop a novel polymer–silicate composite gel system and apply it to mortar specimens to enhance their chloride ion resistance. They combined two key components and testing approaches: (1) the development of the composite gel system by integrating silane with fluorocarbon resin emulsion; and (2) the evaluation of its chloride ion resistance enhancement effect using the RCM method and natural chloride ion penetration tests, coupled with SEM images to analyze the anti-permeation mechanism. Using this approach, Zhang et al. successfully developed the novel polymer–silicate composite gel system and systematically characterized its performance on mortar specimens. The experimental results indicate that the chloride ion migration coefficient of the composite gel system is 4.91 × 10^−12^ m^2^/s, representing a 63.97% reduction compared to the single fluorocarbon gel system, demonstrating a significant improvement in anti-chloride penetration ability. Within the 0–5 mm depth range, the free chloride ion contents at 14, 28, and 56 days decreased by 55.35%, 50.10%, and 43.64%, respectively, further confirming its excellent resistance to chloride penetration. Acid and alkali resistance tests showed that the system retained the inherent corrosion resistance of the fluorocarbon component, ensuring its stability in harsh environments. While carbonation tests demonstrated a slight decrease in carbonation resistance compared with the pure fluorocarbon gel system, the composite gel system still maintained a satisfactory performance level. This polymer–silicate composite gel system represents a significant innovation, as it effectively enhances the mortar’s resistance to chloride ion penetration while retaining key protective properties, overcoming the limitations of single-component gel systems that may lack comprehensive performance. The study provides a proof-of-concept for designing next-generation protective materials for cement-based materials—particularly for concrete structures in chloride-rich environments—where the integration of silane and fluorocarbon resin emulsion enables improved durability and service life, offering valuable technical insights for practical engineering applications.

The paper “Investigation of Toughening Mechanism of Virgin Asphalt by Blending with Waste Battery Powder” explores an innovative application of waste battery powder (WBP) in enhancing the fatigue resistance of sol–gel structured virgin asphalt. The authors develop a WBP-modified asphalt by incorporating a 12% content of WBP, which is designed to address the need for improving pavement service life and achieving resource utilization of waste batteries. While virgin asphalt itself may lack sufficient durability under repeated loading, the WBP modification is intended to enhance its cracking resistance and toughening performance. To clarify this toughening mechanism, the authors conduct linear amplitude, non-damage, and damage time sweep tests to evaluate the fatigue performance of the modified asphalt. They also establish a cracking area model using energy–mechanics balance equations, taking the asphalt cracking area as an index to quantify induced damage and determine the representative rate for the cracking damage process (*k*_cd_). Furthermore, they employ the activation energy for cracking damage (*E*_acd_) to quantify the difficulty of the cracking process. Laboratory test results show that WBP-modified asphalt exhibits three nonlinear cracking stages and has a lower cracking rate compared to virgin asphalt. Its cracking damage shows an overall increasing trend over time, and the damage evolution parameter β can serve as the representative *k*_cd_. Microstructural analysis via scanning electron microscopy reveals that the micro-grooves and wrinkles on the WBP surface enhance its bonding with the asphalt matrix, which significantly increases the *E*_acd_ of sol–gel 70# virgin asphalt from 10.6 to 23.88 kJ·mol^−1^, thereby achieving effective toughening. The study’s findings demonstrate that the unique micromorphology of WBP is the core factor in improving the toughening performance, and the fatigue damage process can be accurately characterized by the kinetic parameters *β* and *E*_acd_. This provides a novel mechanical characterization method for modified asphalt and offers theoretical and technical support for the high-value utilization of waste battery resources in road engineering.

## 3. Conclusions and Outlook

The contributions to this Special Issue highlight the remarkable diversity and innovation in modern polymer gel research, spanning synthesis, characterization, and application across construction, transportation, environment, and biomedicine. Key themes emerging from these studies include the pursuit of sustainability through waste valorization and low-carbon formulations, the development of multifunctional gels with synergistic properties (e.g., self-healing, responsiveness, and durability), and the expansion of application boundaries to address complex engineering and societal challenges.

A critical trend observed is the interdisciplinary integration of polymer gel science with other fields, such as microbiology (EICP technology), nanotechnology (quantum dots, carbon nanotubes), and data science (machine learning models for performance prediction). This cross-disciplinary approach enables the design of advanced gel systems that meet stringent performance requirements while adhering to environmental and economic constraints. Additionally, the emphasis on practical applicability—evidenced by numerous laboratory tests and engineering validation—ensures that these innovations can be translated from the lab to real-world scenarios.

Looking ahead, future research in polymer gels will likely focus on several directions: (1) further optimizing sustainable synthesis routes using bio-based precursors and industrial by-products; (2) developing smart gel systems with real-time responsiveness to multiple stimuli (temperature, pH, stress); (3) enhancing the long-term durability and scalability of gel composites for large-scale engineering applications; and (4) expanding biomedical and sensing applications through improved biocompatibility and sensitivity. Collaboration across academia, industry, and research institutions will be essential to address remaining challenges, such as standardization of performance evaluation, cost reduction, and long-term environmental impact assessment.

In conclusion, this Special Issue showcases the vibrant and evolving nature of polymer gel research, demonstrating its potential to provide innovative solutions to global challenges in sustainability, infrastructure, healthcare, and environmental protection. We anticipate that the findings and insights presented herein will inspire further advancements, foster new collaborations, and drive the development of next-generation polymer gel technologies that are smarter, greener, and more impactful.

## References

[1] Osada Y., Gong J.P. (1998). Soft and Wet Materials: Polymer Gels. Adv. Mater..

[2] Zhang T., Wang K., Wang H., Wei M., Chen Z., Zhong D., Chen Y., Pei P. (2025). Polymer Gels for Aqueous Metal Batteries. Prog. Mater. Sci..

[3] Parvin N., Kumar V., Joo S.W., Mandal T.K. (2024). Cutting-Edge Hydrogel Technologies in Tissue Engineering and Biosensing: An Updated Review. Materials.

[4] Babaei-Ghazvini A., Vafakish B., Patel R., Falua K.J., Dunlop M.J., Acharya B. (2024). Cellulose Nanocrystals in the Development of Biodegradable Materials: A Review on CNC Resources, Modification, and Their Hybridization. Int. J. Biol. Macromol..

[5] Zhang P., Shi B., Dai X., Chen C., Lai C. (2025). A State-of-the-Art Review on the Freeze–Thaw Resistance of Sustainable Geopolymer Gel Composites: Mechanisms, Determinants, and Models. Gels.

[6] Chen Y., Li Z., Guo T., Fang C., Guo P., Wang C., Bai B., Zhang W., Yan H., Chen Q. (2025). Performance and Mechanism Analysis of an Anti-Skid Wear Layer of Active Slow-Release Ice–Snow Melting Modified by Gels. Gels.

[7] Liu C., Lu B. (2025). Self-Healing and Tough Polyacrylic Acid-Based Hydrogels for Micro-Strain Sensors. Gels.

[8] Zhang J., Jia H., Li J., Chen X., Wang L., Wang S., Liu L. (2025). Bio-Gel Formation Through Enzyme-Induced Carbonate Precipitation for Dust Control in Yellow River Silt. Gels.

[9] Correa S., Grosskopf A.K., Lopez Hernandez H., Chan D., Yu A.C., Stapleton L.M., Appel E.A. (2021). Translational Applications of Hydrogels. Chem. Rev..

[10] Nandi A.K., Chatterjee D.P. (2023). Hybrid Polymer Gels for Energy Applications. J. Mater. Chem. A.

